# Maximum standardized uptake value on ^18^F-fluorodeoxyglucose positron emission tomography/computed tomography improves outcome prediction in retroperitoneal liposarcoma

**DOI:** 10.1038/s41598-019-43215-5

**Published:** 2019-04-29

**Authors:** Jinsoo Rhu, Seung Hyup Hyun, Kyung-Han Lee, Sung Jun Jo, Kyo Won Lee, Jae Berm Park, Sung Joo Kim

**Affiliations:** 1 Department of Surgery, Samsung Medical Center, Sungkyunkwan University School of Medicine, Seoul, Korea; 2 Department of Nuclear Medicine, Samsung Medical Center, Sungkyunkwan University School of Medicine, Seoul, Korea

**Keywords:** Sarcoma, Surgical oncology

## Abstract

While ^18^F-fluorodeoxyglucose positron emission tomography/computed tomography (^18^F-FDG PET/CT) has been investigated in extremity sarcomas, there is no evidence on its usefulness in retroperitoneal sarcoma. This study was designed to evaluate the usefulness of ^18^F-FDG PET/CT in predicting aggressiveness of retroperitoneal liposarcoma. Patients experienced surgery for retroperitoneal liposarcoma from November 2007 to February 2018 and underwent preoperative ^18^F-FDG PET/CT were included. Preoperative maximum standardized uptake value (SUV_max_) was calculated. To evaluate the predictability of SUV_max_ for Fédération Nationale des Centres de Lutte Contre le Cancer (FNCLCC) grade 3, receiver operating characteristics (ROC) curve analysis was performed. To analyze whether SUV_max_ can be a risk factor for prognosis, multivariable Cox regression was performed including potential risk factors regarding operation and histopathology. A total of 133 patients were included. ROC curve showed area under the curve of 0.877 (P < 0.001), with a cut-off point of 4.5 SUV_max_ showing 85.7% sensitivity and 78.3% specificity. Cox analyses showed that SUV_max_ > 4.5 was a significant factor for recurrence-free survival (HR = 2.148, CI 1.301–3.546, P = 0.003) and overall survival (HR = 5.052, CI 1.854–13.766, P = 0.002). SUV_max_ is highly predictive of FNCLCC grade 3 and SUV_max_ > 4.5 can be used as a prognostic factor before obtaining the histopathology.

## Introduction

Liposarcoma (LPS) is a mesenchymal origin malignancy that can occur wherever fat is present. LPS typically occurs in the retroperitoneum (up to 40% of cases)^1^, although it can also be found in the mesentery or peritoneum^2^. Besides LPS, other soft tissue sarcomas (STS) including leiomyosarcoma can occur in the retroperitoneum. Although STS accounts for only 1% of all malignancies, it is associated with high mortality and requires specialized management^3^.

Complete surgical resection of retroperitoneal STS is the most important factor related to prognosis^4–7^. However, STS is a highly recurrent disease and its aggressiveness can be well categorized by the Fédération Nationale des Centres de Lutte Contre le Cancer (FNCLCC) grading system^4–8^. Evaluating the aggressiveness of a tumor before treatment may offer clinical advantages with regard to determining the surgical extent and whether neoadjuvant radiation therapy can be an option^9–12^. Computed tomography (CT) has limited value for the evaluation of tumor aggressiveness^13^, and the usefulness of ^18^F-fluorodeoxyglucose positron emission tomography/computed tomography (^18^F-FDG PET/CT) was recently reported in several studies on STS in the extremities^14–17^. ^18^F-FDG PET/CT is frequently used for functional imaging by evaluating tumor glucose metabolism and proliferation^15^. Several studies have reported correlations of hypermetabolic STSs with histopathologic characteristics and their clinical implications for prognosis^18,19^.

However, these previous studies only included limb sarcoma. Since retroperitoneal STSs locate in the deepest region of the abdomen and cause minimum symptoms, they are often discovered incidentally and have an even poorer prognosis than extremity STS. Their close anatomical relationship to vital retroperitoneal structures makes complete surgical resection difficult and has a major impact on survival. Nonetheless, despite the different disease course of retroperitoneal STS compared with extremity STS, the biological characteristics are the same. Therefore, we designed this study to evaluate the usefulness of ^18^F-FDG PET/CT in predicting tumor aggressiveness in retroperitoneal LPS and whether it has clinical implications for prognosis.

## Methods

### Patients

Data on patients who underwent surgery for retroperitoneal LPS from November 2007 to February 2018 were reviewed from the center’s sarcoma database. Among these patients, those who underwent ^18^F-FDG PET/CT before surgery and were proven to have a histopathology of LPS were considered eligible for inclusion. Patients whose FNCLCC grades were unclassifiable were included. Patients who had STS other than LPS were excluded from the study.

### Data collection

Demographic data, treatment history, preoperative CT and ^18^F-FDG PET/CT, operative data, postoperative course, histopathology, and follow-up data regarding recurrence and death were collected. A detailed review of ^18^F-FDG PET/CT images was performed by a nuclear medicine specialist and the preoperative maximum standardized uptake value (SUV_max_) of the tumor was re-evaluated for this study. Medical records were thoroughly reviewed to obtain data on surgical procedures.

### ^18^F-FDG PET/CT imaging and measurement of maximum SUV

All patients fasted for at least 6 hours before the PET/CT study. Blood glucose levels were required to be less than 200 mg/dL. Whole-body PET and unenhanced CT images were acquired using a PET/CT scanner (Discovery STE, GE Healthcare, Waukesha, WI, USA). Whole-body CT was performed using a 16-slice helical CT with 30 to 170 mAs adjusted to the patient’s body weight at a 140-kVp and 3.75-mm section width. After the CT scan, an emission scan was performed from the thigh to the basal skull for 2.5 min per frame in three-dimensional mode 60 minutes after intravenous injection of ^18^F-FDG (5.0 MBq/kg). PET images were reconstructed using CT for attenuation correction with the ordered subsets expectation maximization algorithm (20 subsets, 2 iterations) with matrix of 128 × 128 and voxel size of 3.9 × 3.9 × 3.3 mm. Standardized uptake value (SUV_max_) was normalized to patient body weight.

For measurement of SUV_max_, we placed a spherical volume of interest of 3 cm in diameter at a location where the LPS tissue had highest metabolic activity on PET using the volume viewer software on a GE Advantage Workstation 4.4.

### Data analysis

The study was designed for analysis of two main outcomes. First, we analyzed whether preoperative SUV_max_ can be predictive of FNCLCC grade, which is representative of tumor aggressiveness and has been proven to be related to the prognosis of STS. Second, we analyzed whether a high SUV_max_ can be predictive of the prognosis of the patient.

We performed receiver operating characteristics (ROC) curve analysis for medical diagnostic test evaluation using SUV_max_ of the tumor to predict FNCLCC grade 3 LPS. We also calculated the cut-off point based on the maximum Youden index.

Median SUV_max_ was compared between different histopathologic categories (FNCLCC grade, tumor differentiation, mitotic count, and necrosis) using the Kruskal Wallis test.

To analyze whether the cut-off point of SUV_max_ can be a risk factor for prognosis, survival analyses were performed including data from the first set of PET/CT scans, operation procedures, and histopathology of patients with non-metastatic LPS. Recurrence-free survival and overall survival were analyzed using Kaplan–Meier survival analysis. Potential risk factors for recurrence and death were analyzed using a multivariable Cox proportional hazard model including the cut-off point of SUV_max_. We designed three survival models based on different time points of evaluation. Model 1 contains preoperative variables including the SUV_max_. Model 2 includes operative variables such as specific details of the surgery together with SUV_max_ and other preoperative variables. Model 3 includes the histopathology of the tumor and does not include SUV_max_.

All statistical analyses were performed using SPSS 20.0 (SPSS Inc., Chicago, IL). This study was approved by the institutional review board of Samsung Medical (IRB No. 2018-05-047). The need for informed consent was waived by the institutional review board of Samsung Medical Center due to the retrospective nature of the study. The data can be available on individual investigator’s request.

### Ethical approval

This study was approved by the institutional review board of Samsung Medical (IRB No. 2018-05-047).

### Informed consent

The need for informed consent was waived by the institutional review board of Samsung Medical Center due to the retrospective nature of the study.

## Results

A total of 133 patients met the inclusion criteria of available SUV_max_ data from ^18^F-FDG PET/CT scan, subsequent surgery, and histopathology of LPS. One hundred and nine patients had single data set of PET/CT scans, while 23 patients had two data sets of PET/CT scans after recurrence and 1 patient had three data sets of PET/CT scans after two recurrent episodes. Among these patients, 158 sets of data on PET/CT scan, operation, and histopathology were reviewed.

Table 1 shows the baseline characteristics of the study population. There were 73 males and 60 females with a mean age of 55.9 ± 11.8 years at the initial presentation. Nearly half of the patients (n = 74, 46.8%) had primary LPS while 10.8% and 41.8% had remnant LPS from a previous operation and recurred LPS, respectively. The median SUV_max_ was 3.3 with an interquartile range (IQR) of 4.4. The SUV_max_ of the entire patient population ranged from 0.4 to 41.3. Nearly two-thirds (n = 106, 67.1%) of the PET/CT scans showed a SUV_max_ less than 5. Regarding disease aggressiveness, 40 (25.3%), 80 (50.6%), and 35 (22.2%) patients were classified as FNCLCC grade 1, 2, and 3 respectively. Regarding histology, 37 (23.4%), 113 (71.5%), 7 (4.4%), and 1 (0.6%) patients had well-differentiated LPS (WDLPS), dedifferentiated LPS (DDLPS), myxoid/round cell LPS, and pleomorphic LPS, respectively. Number of mitoses per 10 high power fields was 0–9, 10–19, and ≥20 in 123 (77.8%), 24 (15.2%), and 11 (7.0%) patients, respectively. Necrosis was absent in 73 patients (52.1%), while 62 (44.3%) and 5 (3.6%) patients had <50% and ≥50% necrosis, respectively.

**Table 1:** Demographic, clinical, surgical, and histopathologic data of the study patients.

Factors	No. of patients	%
Patient number	133	
Number of PET/CT and operations performed	158	
Sex, male/female	73/60	54.90%
Mean age (years)	55.9 ± 11.8	
Disease status		
Primary	74	46.8
Remnant	17	10.8
Recurrent	66	41.8
Metastatic	1	0.6
SUV_max_, median (IQR)	3.3 (4.4)	
Minimum-maximum	0.4–41.3	
0–4.9	106	67.1
5–9.9	31	19.6
10–14.9	15	9.5
>15	6	3.8
Anatomy		
Retroperitoneal		
Right		
Suprarenal	5	3.2
Infrarenal	15	9.5
Perirenal	45	28.5
Pelvis	6	3.8
Left		
Suprarenal	3	1.9
Infrarenal	11	7
Perirenal	34	21.5
Pelvis	7	4.4
Intraperitoneal	32	20.3
Histology		
FNCLCC grade		
G1	40	25.3
G2	80	50.6
G3	35	22.2
missing	3	1.9
Tumor differentiation		
WDLPS	37	23.4
DDLPS	113	71.5
Myxoid/round cell LPS	7	4.4
Pleomorphic LPS	1	0.6
Mitosis count, (per 10 HPF)		
0–9	123	77.8
10–19	24	15.2
≥20	11	7
Necrosis		
Absent	73	52.1
<50%	62	44.3
≥50%	5	3.6

### SUV_max_ based on histopathology

Kruskal Wallis test showed significant differences in SUV_max_ between different pathologic features. FNCLCC grade 1, 2, and 3 had median SUV_max_ of 2.25 (IQR 1.5), 3.3 (IQR 2.7), and 9.1 (IQR 8.4), respectively (P < 0.001). WDLPS, myxoid/round cell LPS, and DDLPS had median SUV_max_ of 2.20 (IQR 1.7), 2.8 (IQR 4.2), and 4.3 (IQR 5.4), respectively (P < 0.001). Cases with mitotic count 0–9, 10–19, and ≥20 per 10HPF had median SUV_max_ of 3.0 (IQR 2.7), 5.35 (IQR 7.1), and 13.3 (IQR 9.4), respectively (P < 0.001). Cases with no necrosis, <50% necrosis, and ≥50% necrosis had median SUV_max_ of 2.8 (IQR 2.1), 5.25 (IQR 6.1), and 5.4 (IQR 5.7), respectively (P < 0.001).

### ROC curve of SUV_max_ predicting FNCLCC grade 3

**Figure 1:**
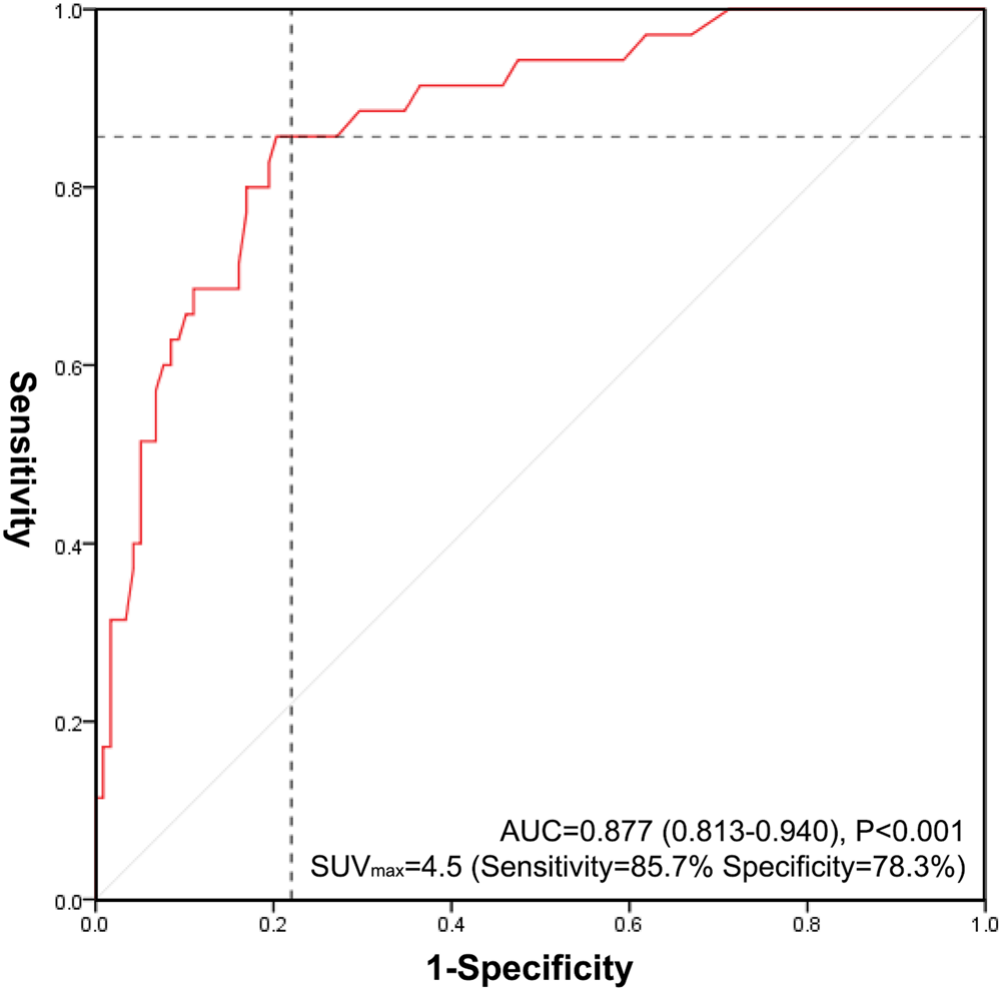
ROC curve for prediction of FNCLCC grade 3 by SUV_max_.

Figure 1 shows the ROC curve of SUV_max_ predicting FNCLCC grade 3. The area under the curve (AUC) was calculated to be 0.877 (confidence interval [CI] 0.813–0.940, P < 0.001). By setting the cut-off point as SUV_max_ of 4.5, sensitivity and specificity were calculated to be 85.7% and 78.3%, respectively.

Table 2 shows the comparisons of characteristics between the SUV_max_ ≤ 4.5 group and SUV_max_ > 4.5 group. There were no differences in sex (P = 0.623), age (P = 0.362), disease status (P = 0.920), and median tumor size (P = 0.514). However, linear-by-linear association analysis showed a significant difference in the distribution of cases according to FNCLCC grade (P < 0.001). There were only five cases with FNCLCC grade 3 in the SUV_max_ ≤ 4.5 group whereas more than half (n = 30, 53.6%) of the SUV_max_ > 4.5 group were FNCLCC grade 3 and there were no grade 1 cases. Differentiation (P < 0.001), mitosis (P < 0.001), and necrosis (P < 0.001) were significantly unfavorable in the SUV_max_ > 4.5 group.

**Table 2:** Comparison of demographic, medical, and histopathologic data between SUV_max_ ≤ 4.5 group and SUV_max_ > 4.5 group.

	SUV_max_ ≤ 4.5 (n = 101)	SUV_max_ > 4.5 (n = 57)	P
Sex, male/female	59/42	31/26	0.623
Mean age (years)	55.6 ± 12.2	57.2 ± 10.3	0.362
Disease status (primary vs. other)			0.920
Primary	47 (46.5%)	27 (47.4%)	
Remnant	14 (13.9%)	3 (5.3%)	
Recurrent	40 (39.6%)	26 (45.6%)	
Metastatic	—	1 (1.8%)	
Median size (cm)	16 (20)	14 (12)	0.514
FNCLCC grade			<0.001
G1	38 (38.4%)	2 (3.6%)	
G2	56 (56.6%)	24 (42.9%)	
G3	5 (5.0%)	30 (53.6%)	
Differentiation (WD vs. other)			<0.001
WDLPS	35 (34.7%)	2 (3.5%)	
DDLPS	60 (59.4%)	53 (93.0%)	
Myxoid/round cell	6 (5.9%)	1 (1.8%)	
Pleomorphic	—	1 (1.8%)	
Mitosis (/10 HPF)			<0.001
0–9	91 (90.1%)	32 (56.1%)	
10–19	8 (7.9%)	16 (28.1%)	
≥20	2 (2.0%)	9 (15.8%)	
Necrosis			<0.001
Absent	58 (67.4%)	15 (27.8%)	
<50%	27 (31.4%)	35 (64.8%)	
≥50%	1 (1.2%)	4 (7.4%)	

### SUV_max_ > 4.5 as a prognostic factor of recurrence-free survival

Survival analyses were based on the first surgical procedure performed in our center for each patient. The 1-, 3-, and 5-year recurrence-free survivals were 64.9%, 43.2%, and 28.1%, respectively. Multivariable Cox regression analyses were performed for three different models. Potential risk factors for recurrence in the univariable analyses were used for multivariable adjusted models. (Table 3)

**Table 3:** Three separate multivariable Cox proportional hazard models analyzing risk factors for recurrence-free survival.

		Recurrence-free survival											
		Univariable			Multivariable								
					Model 1			Model 2			Model 3		
Variables	No	HR	95% CI	P	HR	95% CI	P	HR	95% CI	P	HR	95% CI	P
Male (vs. female)	73	1.764	1.061–2.932	0.029	1.406	0.831–2.379	0.204	1.725	1.030–2.889	0.038	1.895	1.099–3.268	0.021
Remnant or recurrent	55	3.026	1.835–4.991	<0.001	2.590	1.534–4.372	<0.001	2.478	1.490–4.122	<0.001	2.317	1.381–3.888	0.001
SUVmax >4.5	48	2.509	1.535–4.103	<0.001	2.183	1.257–3.793	0.006	2.148	1.301–3.546	0.003		Not included	
Perinephric location	76	0.513	0.317–0.832	0.007	0.514	0.300–0.882	0.016			NS			NS
Suspected invasion	77	1.796	1.077–2.993	0.025	1.824	1.003–3.319	0.049			NS			NS
Gross incomplete resection	42	4.188	2.516–6.970	<0.001		Not included		3.233	1.909–5.474	<0.001	2.941	1.696–5.102	<0.001
Differentiation				0.001		Not included			Not included				0.030
WDLPS	36												
Other LPS	94	3.052	1.610–5.784								2.241	1.082–4.639	
FNCLCC G1/G2	102			<0.001		Not included			Not included				0.014
G3	29	3.223	1.924–5.399								2.117	1.161–3.860	

*Model 1 is modeled based on preoperative variables while model 2 and 3 added operative variables and tumor histopathology to model 1, respectively. Age >60 (P = 0.954), multiplicity (P = 0.635), size >20 cm (P = 0.272), nephrectomy (P = 0.052) and resection of three or more organs (P = 0.424) were excluded from multivariable adjusted modeling due to an insignificant relationship in the univariable analysis.

Model 1 was composed of preoperative variables including SUV_max_ > 4.5. Multivariable Cox analysis showed that SUV_max_ > 4.5 (hazard ratio [HR] = 2.183, CI 1.257–3.793, P = 0.006), remnant or recurrent LPS (HR = 2.590, CI 1.534–4.372, P < 0.001), perinephric location (HR = 0.514, CI 0.300–0.882, P = 0.016), and suspected invasion on preoperative radiology (HR = 1.824, CI 1.003–3.319, P = 0.049) were related to recurrence-free survival.

Model 2 used preoperative variables including SUV_max_ > 4.5 and operative variable. Multivariable Cox analysis showed that SUV_max_ > 4.5 (HR = 2.148, CI 1.301–3.546, P = 0.003), male sex (HR = 1.725, CI 1.030–2.889, P = 0.038), remnant or recurrent LPS (HR = 2.478, CI 1.490–4.122, P < 0.001), and gross incomplete resection (HR = 3.233, CI 1.909–5.474, P < 0.001) were related to recurrence-free survival.

Model 3 added histopathology but excluded SUV_max_ > 4.5. The multivariable model showed that male sex (HR = 1.895, CI 1.099–3.268, P = 0.021), remnant or recurrent LPS (HR = 2.317, CI 1.381–3.888, P = 0.001), gross incomplete resection (HR = 2.941, CI 1.696–5.102, P < 0.001), LPS other than WDLPS (HR = 2.241, CI 1.082–4.639, P = 0.030), and FNCLCC grade 3 (HR = 2.117, CI 1.161–3.860, P = 0.014) were related to recurrence-free survival.

### SUVmax > 4.5 as a prognostic factor of overall survival

The 1-, 3-, and 5-year overall survival rates were 95.5%, 80.9%, and 75.2%, respectively. Multivariable Cox regression analyses were performed for three different models following the principle described above (Table 4).

**Table 4:** Three separate multivariable Cox proportional hazard models analyzing risk factors for overall survival.

		Overall survival											
		Univariable			Multivariable								
					Model 1			Model 2			Model 3		
Variables	No	HR	95% CI	P	HR	95% CI	P	HR	95% CI	P	HR	95% CI	P
Male (vs. female)	73	3.327	1.103–10.034	0.033	3.177	1.051–9.605	0.041			NS			NS
SUVmax >4.5	48	5.642	2.124–14.987	0.001	5.441	2.055–14.411	0.001	5.052	1.854–13.766	0.002		Not included	
Nephrectomy	77	0.288	0.109–0.760	0.012		Not included				NS			NS
Gross incomplete resection	42	7.537	2.698–21.057	<0.001		Not included		6.347	2.238–18.001	0.001	6.415	2.023–20.346	0.002
FNCLCC G1/G2	102			0.002		Not included		—	—	—			0.029
G3	29	4.611	1.758–12.096								3.023	1.119–8.167	

*Model 1 is modeled based on preoperative variables while model 2 and 3 added operative variables and tumor histopathology to model 1, respectively. Age >60 (P = 0.738), remnant or recurrent (P = 0.114), suspected invasion (P = 0.078), multiplicity (P = 0.540), size >20 cm (P = 0.717), perinephric location (P = 0.258), resection of three or more organs (P = 0.073), and differentiation (P = 0.377) were excluded from the multivariable adjusted modeling due to insignificant relationship in the univariable analysis.

Model 1, which included preoperative variables including SUV_max_ > 4.5, showed that SUV_max_ > 4.5 (HR = 5.441, CI 2.055–14.411, P = 0.001) and male sex (HR = 3.177, CI 1.051–9.605, P = 0.041) were related to overall survival.

Model 2, which added operative variables to model 1, showed that SUVmax > 4.5 (HR = 5.052, CI 1.854–13.766, P = 0.002) and gross incomplete resection (HR = 6.347, CI 2.238–18.001, P = 0.001) were related to overall survival.

**Figure 2:**
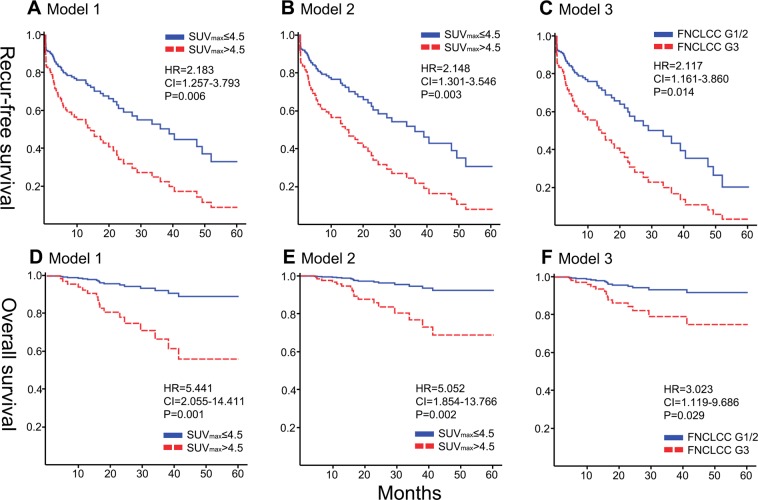
Survival curves analyzed with multivariable Cox proportional hazard models. (**A**–**C**) SUV_max_ > 4.5 was a significant risk factor for recurrence in models 1 and 2 (**A**,**B**) whereas FNCLCC grade 3 was a significant risk factor for recurrence in model 3 (**C**). (**D–F**) SUV_max_ > 4.5 was a significant risk factor for survival in models 1 and 2 (**D**,**E**) whereas FNCLCC grade 3 was a significant risk factor for survival in model 3 (**F**). Model 1 is modeled based on preoperative variables while model 2 and 3 added operative variables and tumor histopathology to model 1, respectively.

Model 3, which added histopathology but excluded SUV_max_ > 4.5 from model 2, showed that gross incomplete resection (HR = 6.415, CI 2.023–20.346, P = 0.002), and FNCLCC grade 3 (HR = 3.023, CI 1.119–8.167, P = 0.029) were related to overall survival. Figure 2 shows the survival curves according to SUV_max_ > 4.5 in model 1 and model 2, and FNCLCC grade 3 in model 3.

## Discussion

This study analyzed the usefulness of ^18^F-FDG PET/CT in predicting tumor aggressiveness represented by FNCLCC grade and yielded promising results. To date, there are only a few studies evaluating ^18^F-FDG PET/CT for extremity STS^16,20–22^. Furthermore, these studies had a heterogeneous background through inclusion of various STSs. Our study focused only on retroperitoneal LPS, which is the most common STS in the retroperitoneum, and showed a high predictability of SUV_max_ for FNCLCC grade 3 and a significant relationship to recurrence and survival when using a cut-off point of SUV_max_ 4.5.

In our sarcoma center, the goal of surgery is to perform gross complete resection for the purpose of obtaining a negative margin. To achieve this goal, we perform radical nephrectomy in cases with a tumor invading or abutting the perirenal fat. Other organs are resected only when tumor invasion is evident and combined resection is inevitable. Previously, we reported the importance of achieving gross complete resection and performing radical nephrectomy when the tumor is abutting perirenal fat^8,23^. The patients undergo ^18^F-FDG PET/CT preoperatively for metastasis work-up, but it also gives information on the metabolic status of the tumor.

The ^18^F-FDG PET/CT scan showed a variable range of SUV_max_ with a median value of 3.3 (range 0.4–41.3). ROC curve analysis showed an AUC of 0.877, and SUV_max_ > 4.5 showed 85.7% sensitivity and 78.3% specificity. Nearly one-third (36.1%) of cases were above 4.5 SUV_max_, and there were no differences in the baseline characteristics except for tumor histopathology between cases above or below 4.5 SUV_max_. Therefore, setting a cut-off point of 4.5 seems appropriate not only due to the high SUV but also based on the data distribution.

In particular, in multivariable Cox regression analyses SUV_max_ > 4.5 showed a significant relationship to poor recurrence-free survival and overall survival. In analysis using three models, SUV_max_ > 4.5 showed usefulness as a prognostic factor comparable to FNCLCC grade. These results might be useful as they allow surgical oncologists to predict tumor aggressiveness by SUV_max_ even before the surgery. The information can be crucial in planning the extent of resection. Surgical oncologists can plan radical resection with combined organ resection when a tumor is predicted to be aggressive. However, a well-designed study is required for making a conclusion on the topic.

The usefulness of ^18^F-FDG PET/CT in STS has been addressed by numerous studies. In 2005, Schwarzbach *et al*. reported that mean SUV can be a useful prognostic parameter by analyzing 55 STSs of the entire body. In their study, 31.1% (n = 23) of patients had retroperitoneal STS, and LPS accounted for 52.7% (n = 39) of total STSs^17^. In 2013, Choi *et al*. published a study on 91 extremity STSs and reported the usefulness of total lesion glycolysis, showing an AUC of 0.833 for predicting disease progression within 2 years^21^. In 2015, Fendler *et al*. reported that metabolic response by^18^F-FDG PET predicts progression-free survival and time to local and distant progression after neoadjuvant chemotherapy for STS^22^. In their study, half of the patients (n = 36, 49%) had STSs in the abdomen or retroperitoneum, and LPS accounted for 18% (n = 13) of patients. However, no previous study focused on retroperitoneal STS, especially LPS.

There are some limitations that must be overcome in future studies. The study only included retroperitoneal LPS and therefore our results cannot be generalized to the other types of STS. However, retroperitoneal LPS can be distinguished by radiologic findings when evaluated by a radiologist specialized in STS. When a retroperitoneal tumor is suspected to be LPS, the ^18^F-FDG PET/CT scan can add valuable information on the aggressiveness of the tumor.

We only used SUV_max_ as a prognostic tool, although SUV_avg_ or metabolic tumor volume (MTV) were also evaluated for practicality. LPSs are usually giant tumors and there is heterogeneity within the tumor mass regarding aggressiveness. Even if a mass is composed of mainly WDLPS, a single solid portion can be DDLPS with high grade. Consequently, the patient may possess a small but high-grade tumor content. These characteristics are in accordance with ^18^F-FDG PET/CT findings that the most fat-containing portion shows low SUV uptake while a single solid portion can be hypermetabolic. Adjusting SUV_avg_ in a giant tumor can deviate the result because grade, not size, is the most important prognostic factor. Therefore, we concluded that SUV_max_ better reflects the clinical situation.

**Figure 3:**
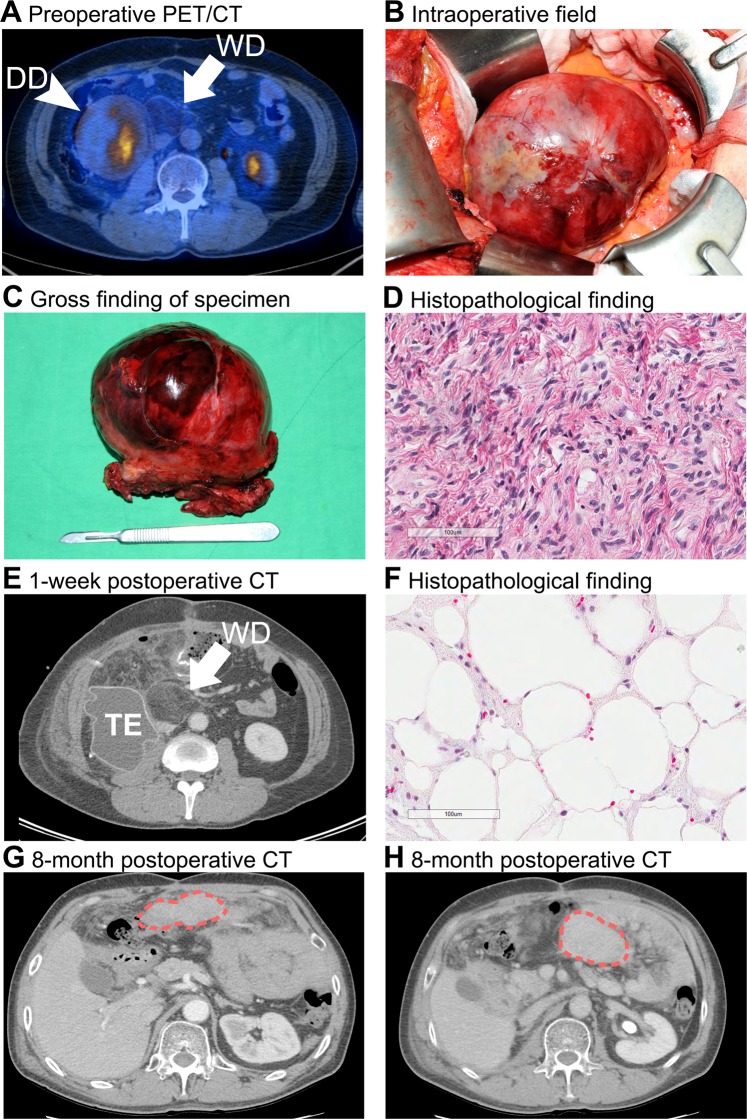
A 56-year-old male patient underwent surgical resection of retroperitoneal LPS. On the preoperative PET/CT scan (**A**), a hypermetabolic solid mass and a fatty mass with no FDG uptake located on the medial side were detected. A solid LPS was resected (**B**) and confirmed as DDLPS with FNCLCC grade 2 (**C**,**D**). However, the 1-week postoperative CT revealed remnant fatty mass on the medial side (**E**). After reoperation, the fatty mass was confirmed as WDLPS, FNCLCC grade 1 tumor (**F**). CT scan performed 8 months after the initial operation revealed peritoneal carcinomatosis with a relatively solid mass (**G**,**H**). The patient died 4 months later. Arrowhead indicates DDLPS, arrow indicates WDLPS, red dotted line indicates solid tumor with peritoneal carcinomatosis. **DD** dedifferentiated liposarcoma, **WD** well-differentiated liposarcoma, **TE** tissue expander.

Figure 3 shows an example of different aggressiveness within a mass. A 56-year old male patient underwent mass excision for retroperitoneal LPS. Preoperatively, the patient had a LPS with a solid portion that showed hypermetabolic features (SUV_max_ = 5.0) and a fatty mass on the medial side with small FDG uptake (SUV = 1.8). During the first operation the solid mass was completely removed and histopathology revealed DDLPS with FNCLCC grade 2. However, the 1-week postoperative CT scan showed that a mass similar to normal fat remained on the medial side near the inferior vena cava. After the second operation, the fatty mass was found to be WDLPS with FNCLCC grade 1. Unfortunately, on the 8-month postoperative CT scan the patient was discovered to have a peritoneal carcinomatosis with several solid masses in the entire abdomen. The patient died 4 months later. This is a typical case that shows the heterogeneous characteristics of a mass in a single patient showing a spectrum from low-grade to high-grade tumor. The small but high FDG-uptake spot that showed SUV_max_ of 5.0 can represent the patient’s prognosis more precisely than the bulk WDLPS with no uptake. For this reason, we consider SUV_max_ to be the key indicator of prognosis in patients with retroperitoneal LPS.

In cases similar to Fig. 3, the clinical significance of preoperative needle biopsy can be limited. In a huge mass, a single site biopsy cannot represent the tumor’s aggressiveness properly. Measuring the SUV_max_ can give valuable information in those cases.

This study including the largest cases of retroperitoneal LPS to date showed that SUV_max_ is highly predictive of FNCLCC grade 3 (AUC = 0.877), and SUV_max_ > 4.5 can be used as a key prognostic factor before obtaining the histopathology report. For clinicians, this can provide vital guidance in planning the treatment strategies for the patient. For example, based on the SUV_max_ of retroperitoneal sarcoma suspected to be liposarcoma, surgeons can plan whether to perform radical resection such as compartmental resection or to preserve adjacent organs while taking effort to obtain gross complete resection. For radiologists, this study provides important guidance for investigating the usefulness of ^18^F-FDG PET/CT in other type of STSs.

## References

[1] Das Gupta, T. K. Tumors and tumor-like conditions of the adipose tissue. *Current problems in surgery*, 1–60 (1970). 4190913

[2] Mentzel T, Fletcher CD (1995). Lipomatous tumours of soft tissues: an update. Virchows Archiv: an international journal of pathology.

[3] Siegel R, Naishadham D, Jemal A (2012). Cancer statistics, 2012. CA: a cancer journal for clinicians.

[4] Bonvalot S (2009). Primary retroperitoneal sarcomas: a multivariate analysis of surgical factors associated with local control. Journal of clinical oncology: official journal of the American Society of Clinical Oncology.

[5] Gronchi A (2009). Aggressive surgical policies in a retrospectively reviewed single-institution case series of retroperitoneal soft tissue sarcoma patients. Journal of clinical oncology: official journal of the American Society of Clinical Oncology.

[6] Gronchi A (2013). Outcome prediction in primary resected retroperitoneal soft tissue sarcoma: histology-specific overall survival and disease-free survival nomograms built on major sarcoma center data sets. Journal of clinical oncology: official journal of the American Society of Clinical Oncology.

[7] Toulmonde M (2014). Retroperitoneal sarcomas: patterns of care in advanced stages, prognostic factors and focus on main histological subtypes: a multicenter analysis of the French Sarcoma Group. Annals of oncology: official journal of the European Society for Medical Oncology/ESMO.

[8] Rhu J (2017). Single-center experience with intra-abdominal liposarcoma: Optimal minimum duration for postoperative remnant tumor screening. Medicine.

[9] Jones JJ (2002). Initial results of a trial of preoperative external-beam radiation therapy and postoperative brachytherapy for retroperitoneal sarcoma. Ann Surg Oncol.

[10] Kirane A, Crago AM (2016). The importance of surgical margins in retroperitoneal sarcoma. J Surg Oncol.

[11] Pasquali S, Gronchi A (2017). Neoadjuvant chemotherapy in soft tissue sarcomas: latest evidence and clinical implications. Ther Adv Med Oncol.

[12] Pisters PW (2004). Phase I trial of preoperative doxorubicin-based concurrent chemoradiation and surgical resection for localized extremity and body wall soft tissue sarcomas. J Clin Oncol.

[13] Chouairy CJ, Abdul-Karim FW, MacLennan GT (2007). Retroperitoneal liposarcoma. The Journal of urology.

[14] Benz MR (2008). Combined assessment of metabolic and volumetric changes for assessment of tumor response in patients with soft-tissue sarcomas. Journal of nuclear medicine: official publication, Society of Nuclear Medicine.

[15] Benz MR (2009). Utilization of positron emission tomography in the management of patients with sarcoma. Current opinion in oncology.

[16] Fuglo HM, Jorgensen SM, Loft A, Hovgaard D, Petersen MM (2012). The diagnostic and prognostic value of (1)(8)F-FDG PET/CT in the initial assessment of high-grade bone and soft tissue sarcoma. A retrospective study of 89 patients. European journal of nuclear medicine and molecular imaging.

[17] Schwarzbach MH (2005). Prognostic significance of preoperative [18-F] fluorodeoxyglucose (FDG) positron emission tomography (PET) imaging in patients with resectable soft tissue sarcomas. Annals of surgery.

[18] Macpherson RE (2018). Retrospective audit of 957 consecutive (18)F-FDG PET-CT scans compared to CT and MRI in 493 patients with different histological subtypes of bone and soft tissue sarcoma. Clin Sarcoma Res.

[19] Skamene SR (2014). Metabolic activity measured on PET/CT correlates with clinical outcomes in patients with limb and girdle sarcomas. J Surg Oncol.

[20] Andreou D (2014). Prognostic relevance of (1)(8)F-FDG PET uptake in patients with locally advanced, extremity soft tissue sarcomas undergoing neoadjuvant isolated limb perfusion with TNF-alpha and melphalan. European journal of nuclear medicine and molecular imaging.

[21] Choi ES (2013). Total lesion glycolysis by 18F-FDG PET/CT is a reliable predictor of prognosis in soft-tissue sarcoma. European journal of nuclear medicine and molecular imaging.

[22] Fendler WP (2015). PET response criteria in solid tumors predicts progression-free survival and time to local or distant progression after chemotherapy with regional hyperthermia for soft-tissue sarcoma. Journal of nuclear medicine: official publication, Society of Nuclear Medicine.

[23] Rhu, J. *et al*. Radical Nephrectomy for Primary Retroperitoneal Liposarcoma Near the Kidney has a Beneficial Effect on Disease-Free Survival. *World journal of surgery* (2017). 10.1007/s00268-017-4157-6 28808758

